# Computer-Assisted Design of Peptide-Based Radiotracers

**DOI:** 10.3390/ijms24076856

**Published:** 2023-04-06

**Authors:** Vincenzo Patamia, Chiara Zagni, Ilaria Brullo, Erika Saccullo, Alessandro Coco, Giuseppe Floresta, Antonio Rescifina

**Affiliations:** Dipartimento di Scienze del Farmaco e della Salute, Università di Catania, Viale A. Doria 6, 95125 Catania, Italy; vincenzo.patamia@unict.it (V.P.); chiara.zagni@unict.it (C.Z.);

**Keywords:** imaging, nuclear medicine, peptide probes, computer-assisted molecular design, CADD

## Abstract

In medical imaging, techniques such as magnetic resonance imaging, contrast-enhanced computerized tomography, positron emission tomography (PET), and single-photon emission computed tomography (SPECT) are extensively available and routinely used for disease diagnosis. PET probes with peptide-based targeting are typically composed of small peptides especially developed to have high affinity and specificity for a range of cellular and tissue targets. These probes’ key benefits include being less expensive than traditional antibody-based PET tracers and having an effective chemical modification process that allows them to be radiolabeled with almost any radionuclide, making them highly appealing for clinical usage. Currently, as with every pharmaceutical design, the use of in silico strategies is steadily growing in this field, even though it is not part of the standard toolkit used during radiopharmaceutical design. This review describes the recent applications of computational design approaches in the design of novel peptide-based radiopharmaceuticals.

## 1. Introduction

Radioactive isotopes of certain elements are known as radionuclides and can be used for cancer treatment and medical imaging [1]. More than 20 years ago, the somatostatin receptor-targeted ligand DOTATOC received approval for the first time for diagnostic and therapeutic uses in patients with somatostatin-expressing neuroendocrine tumors. This ligand can be labeled with various threefold charged cations, resulting in compounds with increased somatostatin receptor affinity [2]. This approval paved the way for other clinical uses of analogs with chemical structures similar to peptides [3]. Since the approval of DOTATOC, impressive improvements have been made in developing novel radiopharmaceuticals for theranostic applications.

Positron emission tomography (PET) and single-photon emission computed tomography (SPECT) are widely used imaging modalities that can detect radioactive signals from radiolabeled probes. These diagnostic techniques offer the opportunity to produce exceptional new diagnostic and therapeutic radiopharmaceuticals. In fact, in the last few years, the computational design of peptide-based PET and SPECT imaging probes has gained significant attention since it allows for the rational design of probes with improved properties, such as higher binding affinity, selectivity, and pharmacokinetics. Unfortunately, due to the requirement for specially trained staff, time-consuming research protocols, and the amount of time needed on highly specialized, functional animal imaging systems, the design of radiopharmaceuticals necessitates significant investments throughout the research phase. Designing new radiotracers that meet these criteria is incredibly difficult, time-consuming, and costly. A new and popular tool, namely computer-aided drug design (CADD), can be employed to overcome these limitations. It can play an essential role in developing therapeutically important molecules, and it is considered very useful, at least for preclinical application and in the drug design phase [4,5,6]. CADD approaches offer insights into protein–ligand binding interactions and important chemical properties to lead to the identification and development of high-affinity radioligands [7].

Peptide-based imaging probes have several advantages over small-molecule probes, including higher target specificity and lower toxicity. Peptides typically comprise a few to several dozen amino acids, allowing for more diverse sequences and three-dimensional structures. This diversity enables peptides to target various biomolecules, including enzymes, receptors, transporters, and antigens, in disease pathogenesis. Furthermore, peptides can be chemically modified [8] to improve their pharmacokinetic properties, resulting in higher stability [9,10] and lower immunogenicity [11,12] than the antibody-like compounds. The ability of all biological medications to trigger an immune response in the host, known as immunogenicity, is currently well understood. This includes pharmaceuticals with a human structure, i.e., even a human amino acid sequence could trigger the immune system. Patients with compromised immune systems, a patient’s genetic background, the administration route, contaminants, the structure of the protein homolog, and other unidentified variables can all affect how immunogenic biological agents behave [13].

PET and SPECT imaging rely on the use of radioactive isotopes. To exploit these peptides as imaging or therapeutic agents, they must be covalently or non-covalently radiolabeled with selected radioactive isotopes [1,14].

These probes allow focused diagnostic and targeted therapy in several fields, such as cardiology, endocrinology, and neurology, to investigate infection, inflammation, and, most importantly, oncology. This field of application is particularly relevant because of the rising global frequency of malignant tumors in the population. The interactions of the radiolabeled peptide-based probe with cancer cells are typically carried out through protein structures that are overexpressed in these malignant cells [14,15,16,17,18,19]. These radioactive isotopes can be attached to peptides to create imaging probes that bind to specific molecular targets in vivo. Several computational strategies can be employed to design peptide-based PET and SPECT imaging probes. This review will discuss the more recent applications of computational design strategies of peptide-based PET and SPECT imaging probes.

## 2. Discussions

Our journey in the report of the most recent application of CADD in peptide-based radiotracers starts with a contemporary application of the molecular docking analyses performed by Kim et al. [20]. In this study, in silico investigation has been used to identify the overproduction of a specific protein responsible for Alzheimer’s disease. Alzheimer’s disease (AD) is characterized by abnormally accumulated Aβ42, which is prone to aggregating into senile plaques that may develop into oligomers. These oligomers can pass through the cerebral–cerebrospinal fluid (CSF) barrier, decreasing their concentration in the brain. A reduced Aβ42 concentration in the CSF can be a sign of AD. Generally, Aβ42 has been detected in numerous experiments using antibodies for application in many diagnostic assays, such as ELISA [21]. Moreover, this method is limited because it cannot detect low-molecular-weight amyloid aggregates. An Aβ42 detection system was developed using a peptide as an imaging PET probe to solve this problem. It was possible to identify the peptide moieties that bound most strongly to the monomeric state of Aβ42; the binding sites were also estimated using molecular docking analyses.

**Figure 1:**
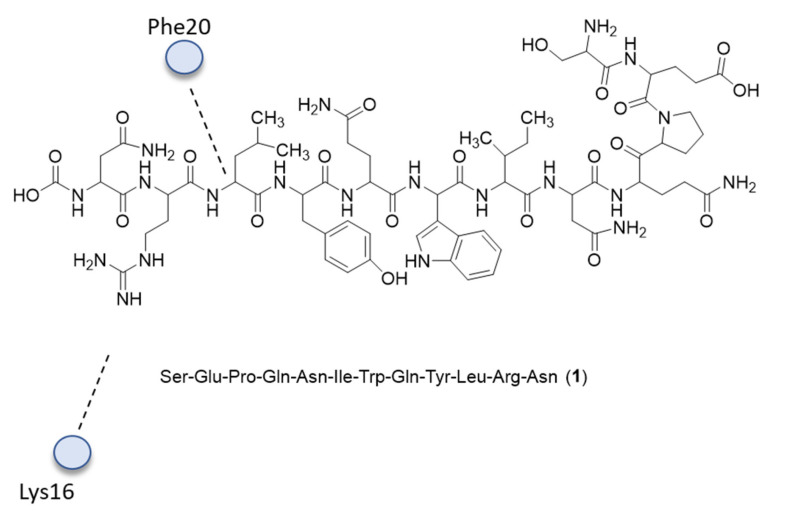
Structure of Aβ42 binding with compound **1**. Only the most relevant calculated interactions are shown.

Biopanning [22] was used as a screening method [23,24] to select the Aβ binding peptide probe (ABPP) sequences. After four rounds of biopanning performed in different conditions, each screened phage presenting its peptide sequence was analyzed, and the best one was selected. To predict noncovalent interactions between the selected peptide and Aβ42 (PDB ID: 1Z0Q) and estimate the peptide binding site, molecular docking analysis was performed using AutoDock Vina. The flexible and non-flexible residues were identified, and the relevant charges were added to calculate a more accurate ligand–protein interaction. Free torsional bonds were modeled into the structure of the peptide ligand. Docking analysis showed that molecule **1** (Ser-Glu-Pro-Gln-Asn-Ile-Trp-Gln-Tyr-Leu-Arg-Asn) (Figure 1) binds to the mid-to-end regions of the Aβ42 sequences. Notably, the peptide chains of Arg11 and Leu10 in the screened peptide established hydrogen bonds with the polypeptide chains of Lys16 and Phe20 in Aβ42.

In addition, Ile31, Ile32, Gly33, and Leu34 in Aβ42 also interact with the target peptide **1**, forming hydrogen bonds with Ser1, Glu2, and Trp7 residues.

**Figure 2:**
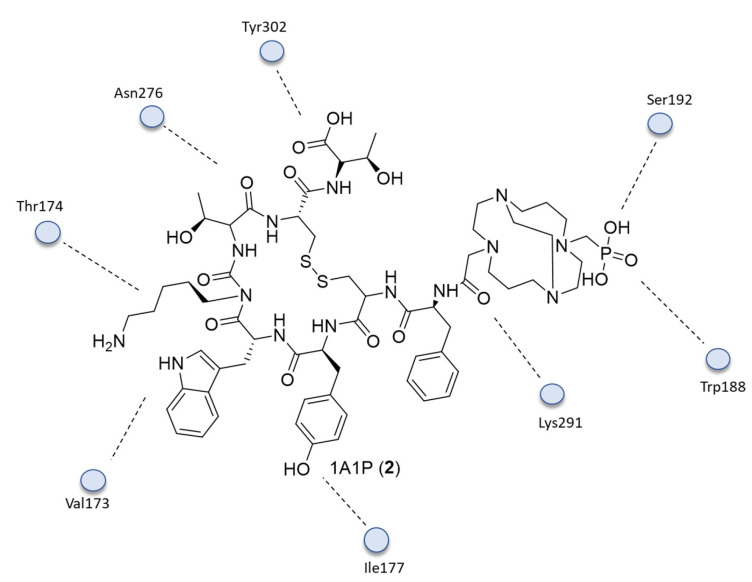
The 2D interactions of **2**.

In another study, docking was performed by the Tripos Surflex-Dock [25] module with different binding poses of a selected ligand and SSTR2, one of the five subtypes of somatostatin receptors overexpressed in many neuroendocrine tumors [26]. The binding pocket was rendered with the molecular surface generated by Sybyl MOLCAD. The docking poses and ligand–receptor interactions were analyzed via the PyMol software. Docking calculations were first performed on Tyr3-octreotate (Y3-TATE), proving that this ligand was deeply allocated within the receptor. At the same time, an empty pocket next to Y3-TATE’s *N*-terminus was still present, indicating that the protein should tolerate substitution well at this site. In addition, hydrogen bond interactions of Y3-TATE with amino acid residues of SSTR, such as Asn196, Trp197, Tyr205, Asn276, Asn119, Gln88, and Thr212, were shown. Utilizing the information from the docking data (i.e., by conjugating them to the *N*-terminus), several chelators were conjugated to Tyr3-octreotate to form CB-TE1A1P−Y3-TATE (1A1P, **2**), CBTE1A1P−DBCO−Y3-TATE (AP, **3**), and TE1K1P−PEG4−DBCO−Y3-TATE (KP). Docking calculations were then performed for the modified sequences and highlighted that 1A1P interacts with the amino acid residues Trp188, Ser192, Lys291, Asn276, Tyr302, Thr174, Val173, Ile177, and Phe208 of SSTR2 through hydrogen bonds (Figure 2).

**Figure 3:**
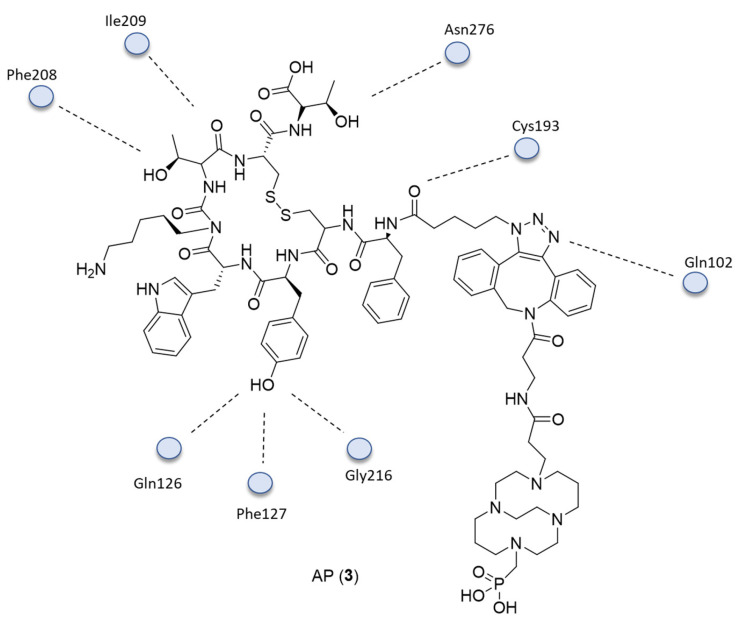
The 2D interactions of **3**.

Additional docking studies conducted to study the interaction of AP and SSTR2 showed the instauration of hydrogen bonds between AP and Asn276, Cly216, Cys193, Gln126, Ile209, Phe127, Phe208, and Gln102 residues (Figure 3).

Finally, the docking of KP with the SSTR2 model protein demonstrated superior stability.

**Figure 4:**
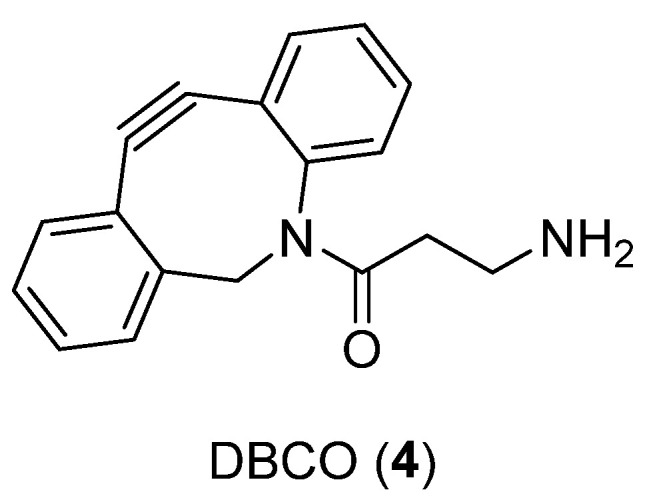
Structure of **4**.

The efficiency of the labeling of the novel generated probe with dibenzocyclooctyne (DBCO, **4**) (Figure 4) as a linker complexed with copper-64 was studied because it is known that DBCO could significantly change the binding affinity, but also the solubility and pharmacokinetics, of the imaging probe. Therefore, modeling was performed to determine whether modifications to the *N*-terminus with the bulkier DBCO group would be tolerated. The in silico docking supported the notion that the modifications were unlikely to cause drastic changes in the binding affinities.

A PEG linker was incorporated into KP to lessen the impact of the hydrophobic DBCO on the tracer’s pharmacokinetics. It showed that KP fits the binding pocket even with the flexible PEG linker between the chelator and DBCO, with an optimal predicted docking score. The peptide interacts with Trp197, Ile195, Thr206, Gly123, Asp122, Asn276, Gln126, Thr212, and Lys291 of SSTR2 through hydrogen bonds. Notably, docking studies revealed a common amino acid residue of SSTR2, Asn276, identified as crucial for SSTR2 affinity. The model and docking method may be useful for future SSTR2 ligand design and structure–activity studies even without a crystal structure, as evidenced by the consistency of the computational docking.

**Figure 5:**
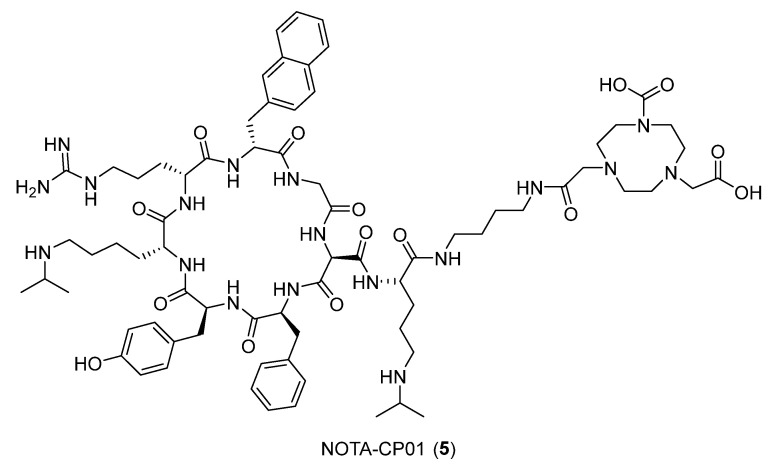

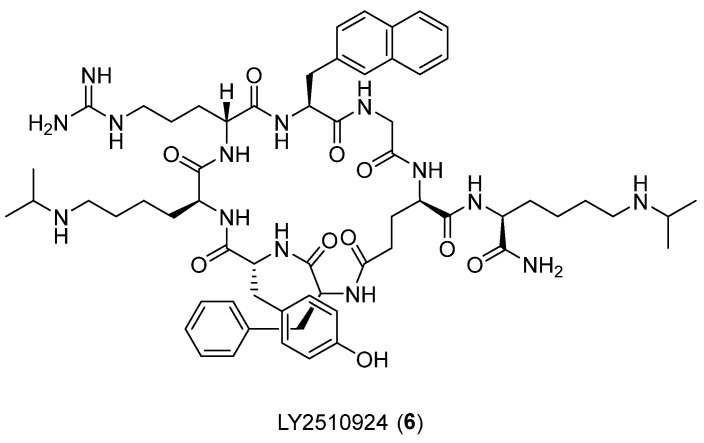
Structures of **5** and **6**.

Based on the evidence that the C-X-C chemokine receptor 4 (CXCR4) is overexpressed in metastatic esophageal squamous cell carcinoma (ESCC), a study conducted by Peng et al. (2021) [27] produced a probe for the imaging of such systems. Figure 5 reports the chemical structures of NOTA-CP01 (cyclo[Phe-Tyr-Lys-(*i*Pr)-d-Arg-2-Nal-Gly-d-Glu]-Lys-(*i*Pr)-butane-1,4-diamine-bis-*t*-butyl NOTA-NH_2_, **5**) and LY2510924 **6**, which is a potent cyclopeptide CXCR4 antagonist developed for the noninvasive visualization of CXCR4 expression in metastatic ESCC.

**Figure 6:**
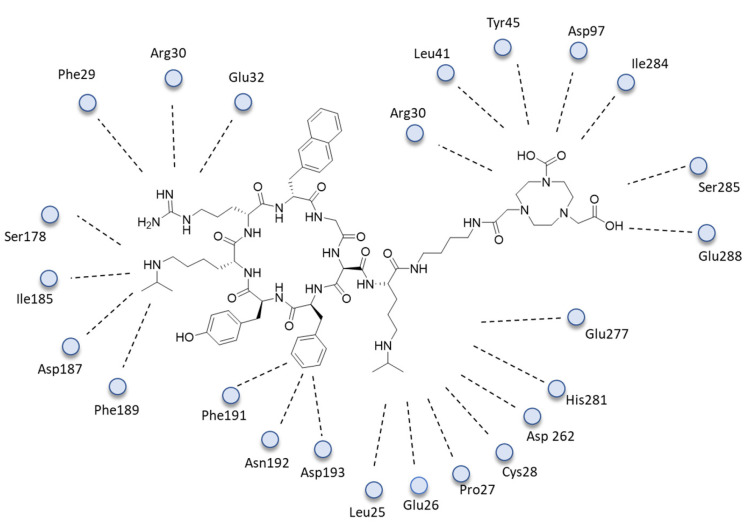
The 2D interactions of CXCR4/5.

The C-X-C chemokine receptor type 4 (CXCR4), a conserved seven-transmembrane structure spanning the G-protein-coupled receptor, was identified as contributing to tumor metastasis in response to its endogenous ligand stromal cell-derived factor 1 (SDF-1, also named CXCL12). The combination CXCR4–CXCL12 has been identified as an attractive therapeutic target, and numerous CXCR4 antagonists, including peptide **5**, have been developed as delivery systems used in PET imaging. Given the established copper coordination chemistry that permits reactivity with a wide range of chelating systems that may be coupled to peptides, the use of ^64^Cu for labeling is advantageous. Building on these advantages, researchers designed a new molecular probe [NOTA-CP01], labeled with copper-64 generated from **6**, that exhibited CXCR4-specific binding with a similar nanomolar affinity. In the designed tracer, the terminus of **6** was modified with a bis-*t*-butyl NOTA chelator via a butane-1,4-diamine linker. The native peptide sequence **6** and the modified one **5** were then studied through molecular modeling experiments. LY2510924 and NOTA-CP01 were prepared by the LigPrep module of Schrödinger 2015 (Schrödinger, LLC, New York, NY, USA, 2015). Finally, **6** and **5** were docked into the binding site of the CXCR4 chemokine receptor complex and evaluated using Glide’s standard precision scoring function. Both molecules showed optimal docking scores. The CXCR4/**5** (Figure 6) complex showed that the core-specific interactions of the ligand were formed by two lysines, an arginine, phenylalanine, and the NOTA. Lys3 made polar interactions with Ser178 (2.4 Å), Ile185 (3.1 Å), Asp187 (2.5 Å), and Phe189 (2.6 Å) and formed a hydrogen bond with Ser178 (2.4 Å). Lys8 made polar interactions with Leu25 (3.4 Å), Glu26 (3.0 Å), Pro27 (3.0 Å), Cys28 (2.9 Å), Asp262 (3.1 Å), Glu277 (1.7 Å), and His281 (2.4 Å) and formed two hydrogen bonds with Glu277 (1.7 Å) and His281 (2.4 Å). Arg4 made polar interactions with Phe29 (3.2 Å), Arg30 (2.3 Å), and Glu32 (1.8 Å) and formed a hydrogen bond with Glu32 (1.8 Å). Phe1 established polar interactions with Phe191 (3.2 Å), Asn192 (3.8 Å), and Asp193 (3.1 Å). NOTA formed polar interactions with Leu41 (3.3 Å), Tyr45 (2.7 Å), Asp97 (3.3 Å), Ile284(3.4 Å), Ser285 (2.9 Å), and Glu288 (2.2 Å) and two hydrogen bonds with Arg30 (2.3 Å), Tyr45 (2.7 Å), and Glu288 (2.2 Å). Additionally, the eighth residue of the ligand filled most of the binding pocket volume in the CXCR4/**5** complex, with the NOTA positioned at the bottom of the binding pocket. As a result, it was shown in this study that noninvasive CXCR4 detection by PET imaging using the ^64^Cu-labeled CXCR4 antagonist LY2510924 is feasible and may represent a suitable method for ESCC diagnosis.

**Figure 7:**
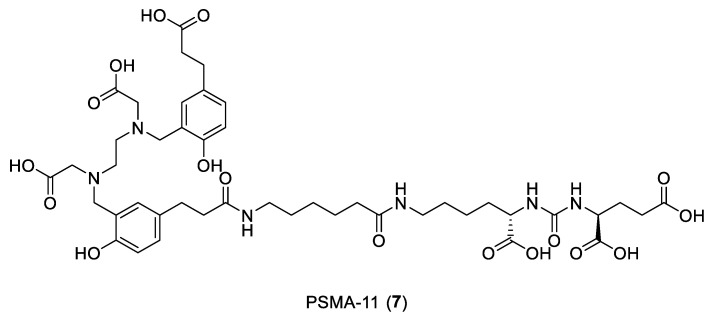

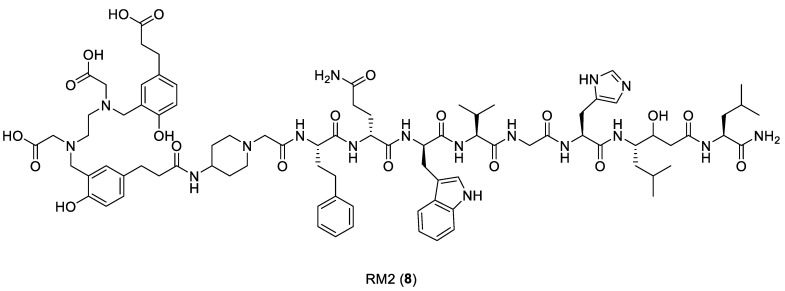
Structures of compounds **7** and **8**.

In the study conducted by Liolios et al. [28], the prostate-specific membrane antigen (PSMA) and gastrin-releasing peptide receptor (GRPR) were considered as valuable biological targets for the molecular imaging and therapy of prostate (PCa) and breast (BCa) cancer. They both are widely expressed on the surfaces of PCa cells and BCa cells during the progression of malignancies. Among them, two new peptides were developed for PET diagnosis: [^68^Ga]Ga-1 or PSMA-11 **7**, in which the Glu-urea-Lys PSMA pharmacophore is attached via the 6-aminohexanoic acid (Ahx) spacer to the chelator HBED-CC (*N*,*N*′-bis-[2-hydroxy-5-(carboxyethyl)benzyl]ethylenediamine-*N*,*N*-diacetic acid), and [^68^Ga]Ga-2 (Y) based on the RM2 structure **8**, which is a GRPR antagonist with the 4-amino-1-carboxymethyl piperidine-[(*R*)-Phe6,Sta13]BN(6-14) peptide portion conjugated to the HBED-CC chelator (Figure 7).

The molecular interactions of the ligands with their receptors were investigated using docking calculations and molecular dynamics (MD) simulations. For the MD analysis, 100 ns of simulations were performed with the Desmond algorithm and OPLS2005 force field [29], using Desmond and a default MD simulation protocol for soluble proteins. For compound RM2, the Prime software was utilized for the alignment and homology modeling of the structure, selecting the “Energy-Based Building Method”, which constructs and refines residues based on their energy rather than their configuration in the template structure. The complex was further processed using the Protein Preparation Workflow (Schrödinger Release 2020-4: Maestro, Schrödinger, LLC, New York, NY, USA, 2020). The docking studies were performed within the binding area of glutamate carboxypeptidase II (GCP II) using the X-ray structure of the Lys-NH-CO-NH-Glu analog urea-based inhibitor (DCIBzL) in its complex with GCP II (PDB ID: 3D7H). The binding cavity for the Lys-NH-CO-NH-Glu pharmacophore group of **8** can be divided, as in all Glu-ureido binders, into the nonprime (S1) and prime (S1′) sections, separated by the active site harboring two Zn^2+^ ions connected to the urea group of the inhibitor. Docking calculations of PSMA-11 to GCP II were performed using ChemScore as the scoring function. The highest-scored docking pose accurately described DCIBzL inside the binding area of GCP II, and both the two highest-scored docking poses for **8** were similar and used for the subsequent 100 ns MD simulations.

**Figure 8:**
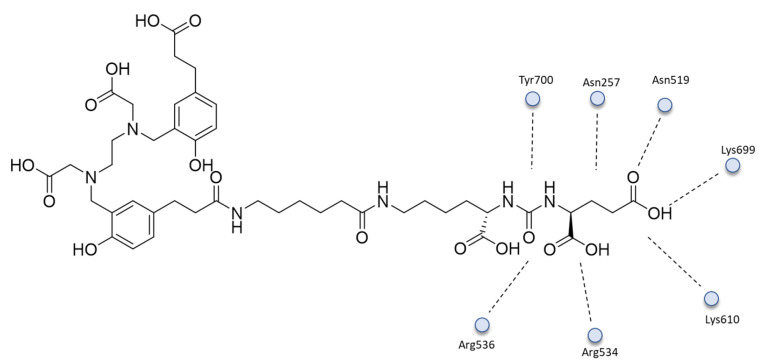
The 2D interactions of **7**.

The MD simulations showed that **7** (Figure 8) forms critical hydrogen-bonding interactions between the lysine and glutamate portions of the peptide with Arg536 and the Arg534, Lys610, Lys699, Asn519, Asn257, and Tyr700 residues of the protein. Moreover, van der Waals interactions between the lysine’s side-chain carbons and the phenyl side chains of Phe209 and Tyr700 are established. The chelating moiety forms ionic hydrogen bonding between its carboxylate groups and Arg511 and Lys610, and a van der Waals interaction between the linker or chelating HBED-CC group and the carbon side chains of Phe546, Arg511, and Arg463 in the arene binding site.

**Figure 9:**
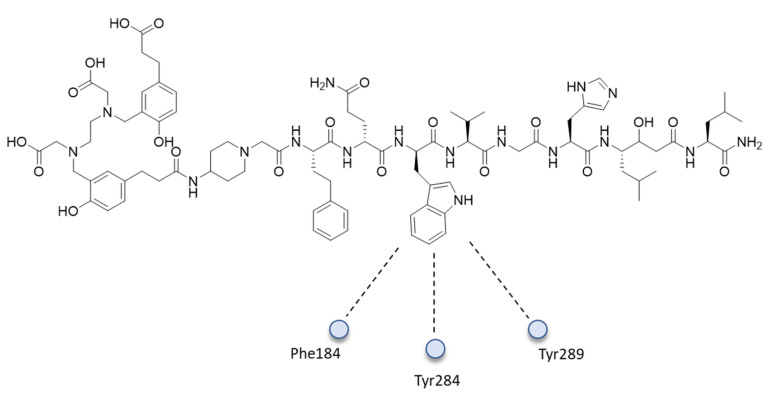
Main hydrophobic interactions of Trp8 with **8**.

GRPR (BB2R) is a class of GPCR in which some peptide ligands—for example, the natural bombesin and its peptide analogs—bind with their *C*-terminal-amidated carboxyl group, which interacts with the helical cavity of GPCR. The BB2R binding antagonist **8** was investigated by MD. The 100 ns MD simulations showed that **8** dives into the BB2R TM helical cavity, forming direct hydrogen-bonding interactions through its amidated end with Gln120^3.32^, Glu175^4.60^, and Ser179^4.64^ and water-mediated hydrogen bonds with Ala197^EL2^ and Pro198^EL2^, while critical water-mediated hydrogen bonds are formed between the backbone peptidic bonds of His11 and Ala9 and the side chains of Arg308^7.39^ and Arg287^6.58^, respectively. The most important hydrophobic interactions are the *π*−*π* stacking between Trp8 of the peptide antagonist and Phe184^EL2^, Tyr284^6.55^, and Tyr289^6.60^ residues (Figure 9).

Moreover, according to the output of the NMR spectroscopy and 4 μs MD simulations, bombesin was found to possess helical structures at the *C*-terminus that tend to unwind at the end of the peptide chain, while the peptide presents a hairpin turn structure at the *N*-terminus. Additionally, mutagenesis studies of the bombesin receptors (BB2 and BB3) indicate the importance of asparagine or arginine for bombesin binding. At the same time, the mutation of tyrosine in BB2 is critical for the activity of the peptide. Three additional polar residues are conserved and essential for peptide binding within the bombesin family. Finally, the results of the biomolecular simulations and MD calculations have provided more insight into the molecular interactions of PSMA and GRPR ligands, which may be essential for developing new ones.

Sialic acid-binding Ig-like lectin 9 (Siglec-9) is a protein belonging to the immunoglobulin superfamily, able to bind vascular adhesion protein-1 (VAP-1), an important biological target. VAP-1’s enzymatic activity regulates the adhesion and exudation of leukocytes in and from blood vessels. In normal conditions, the receptor is missing from the endothelial cell surface and stored inside intracellular granules. From there, it is translocated to the endothelial cell surface under inflammation and certain cancers and, thus, leaves the normal endothelium nontargeted during imaging. This feature makes vascular adhesion protein-1 a suitable and attractive imaging target.

**Figure 10:**
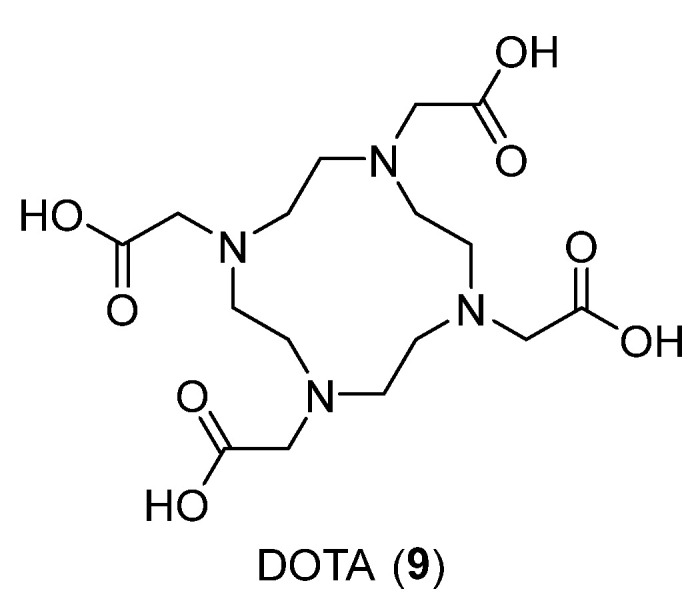
Structure of compound **9**.

In the work reported by K. Aalto et al. [30], they showed the usefulness of a Siglec-9 targeting peptide as an imaging tool for inflammation and cancer in PET imaging. As an imaging agent, the Siglec-9 peptide is conjugated with 1,4,7,10-tetraazacyclododecane-1,4,7,10-tetraacetic acid (DOTA, **9**) and then labeled with ^68^Gallium (Figure 10).

Siglec-9 can be used as a prototype to design a new peptic probe capable of binding VAP-1, and, thanks to the modeling studies, it was possible to identify the binding site for the target.

**Figure 11:**
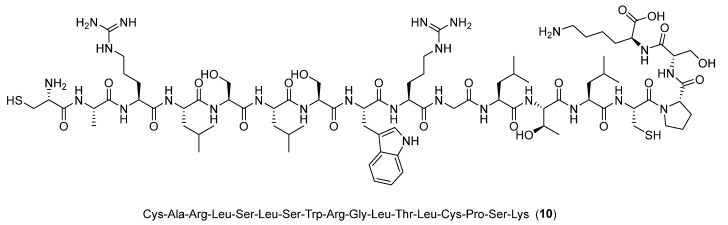
Structure of the peptide **10**.

The cyclic peptide **10**, Cys-Ala-Arg-Leu-Ser-Leu-Ser-Trp-Arg-Gly-Leu-Thr-Leu-Cys-Pro-Ser-Lys (Figure 11), containing residues 283 to 297 from Siglec-9, was generated using SYBYL Version 7.3 (Tripos), based on the structural model of the second Siglec-9 domain, the one implied in the binding process.

The protein domain implied in the binding was determined through modeling studies using the crystal structure of the first Siglec-5 C2 domain (PDB ID: 2ZG2).

Both docking and binding studies agree that the peptide fits the enzymatic pocket of vascular adhesion protein-1 (VAP-1), belonging to the copper/topaquione oxidase family. The best binding poses were achieved when Arg284 was covalently bound to topaquinone (the prosthetic group of the copper-containing amine oxidases). The peptide also contains two arginines, each of which could bind covalently to topaquinone in the VAP-1 structure (PDB ID: 1US1). Both binding patterns were tested to detect which gives the best calculated binding results. Arginine binds covalently to the N5 atom of topaquinone in an active conformation; therefore, different dockings were performed using GOLD Version 3.2. Docking results showed that the highest affinity value was obtained when Arg284 was bound to topaquinone. Arg284 is located on the molecule’s surface and freely accessible to its counter-receptor; however, efficient binding seems to require two arginines (Arg284 and Arg290), one binding to topaquinone and the other one facilitating interaction by binding to another site in the pocket.

**Figure 12:**
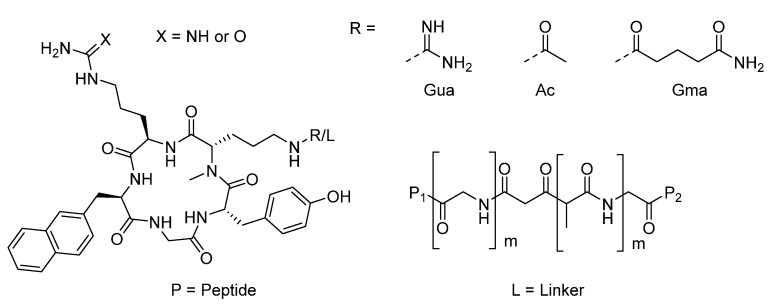
Structures of cyclic pentapeptides.

In the study by O. Demmer et al. [31], a library of monomeric and dimeric cyclic pentapeptides (Figure 12) that bind the chemokine receptor CXCR4 was analyzed through docking analysis. The purpose was to use the peptides as potential radiotracers for the noninvasive molecular detection of primary tumors and targeted metastases.

**Figure 13:**
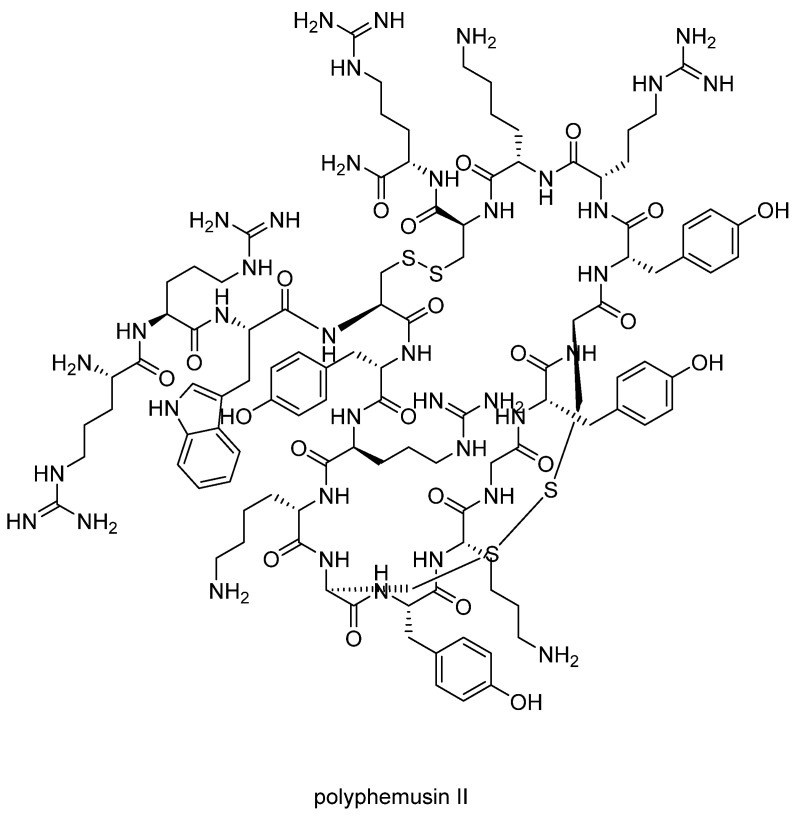
Structure of polyphemusin II.

The targeted protein CXCR4 (PDB: 3OE0) is a critical regulator of inflammation and immune surveillance implicated explicitly in cancer metastasis and HIV-1 infection. Moreover, CXCR4 is overexpressed in more than 70% of cancers, making it an ideal target for personalized treatments. The probes were designed based on the known antagonist ligands of the CXCR4 receptor; all are cyclic analogs of the horseshoe crab peptide polyphemusin II (Figure 13).

Data show that dimerization leads to better affinity for polyphemusin II derivatives since it allows two binding sites, one responsible for the affinity and the other for the signaling function. In addition, it is well known that CXCR4 can form homo- and heterodimers. This could be an alternative explanation for why some symmetric dimers have superior binding properties to their monomers. For this reason, first, it was investigated whether the dimeric compounds could occupy the two binding sites of the receptor dimer. The hypothesis was immediately refuted by measuring the distance between the two sites (≈40 Å), which does not allow the dimer to occupy the binding site. The only other possibility is the existence of two neighboring binding sites in the CXCR4 monomer: site one, located in the extracellular portion, *N*-terminus, and extracellular loops (only partially solved), and site two, located in the fully solved transmembrane bundle region. This is another site for further interaction with aromatic and basic groups addressed by the dimer’s second peptide unit with a more unspecific binding mode. Available data came from docking studies of the cyclic peptide monomer and dimeric peptide interaction with the receptor. The best binding pose suggested by the docking software Glide [32] has the peptide occupying most of the binding pocket. After this, molecular modeling studies were also performed to determine the dimeric compounds’ binding mode. In this regard, the modeling studies started from the assumption that one cyclopentapeptide in compounds would bind site two in the same fashion as the corresponding monomeric form. The built complex was then subjected to a Monte Carlo search of all the energetically feasible conformations of the linker and peptidic portions in the receptor context. This simulation resulted in two prominent conformation families in which the peptide occupying site one alternatively points toward the II extracellular loop and the II transmembrane domain or toward the IV and V transmembrane domains. In both cases, the intrinsic flexibility of the CXCR4 site suggests that this receptor region could plastically be adapted to the different dimeric cyclopentapeptides, depending on their linker length. Additionally, in both suggested conformations, the Arg^3^ residue of the peptide that occupies site one forms Coulombic interactions with negatively charged receptor regions. This would be in line with SAR data, demonstrating that substituting Arg^3^ with l-citrulline leads to a loss in affinity.

**Figure 14:**
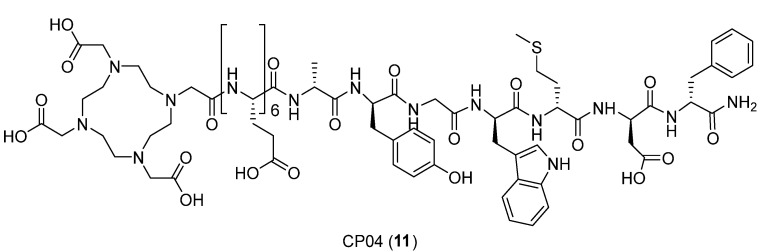
Structure of **11**.

Another important target for conjugates complexed with DOTA and ^68^Ga for diagnostic imaging and targeted radionuclide therapy, studied by Lipinski et al. [33], is the cholecystokinin receptor subtype 2 (CCK-2R). Since CCK-2R is overexpressed on medullary thyroid carcinoma cells (MTC), targeting peptides are useful as a vector for diagnostic imaging and targeted radionuclide therapy (TRNT) of MTC. Several peptide-conjugates suitable for this purpose have been synthesized, such as a very promising minigastrin analog, DOTA-(DGlu)_6_-Ala-Tyr-Gly-Trp-Met-Asp-Phe-NH_2_, called CP04 **11** (Figure 14).

Due to the properties of the macrocyclic chelator DOTA, which is *N*-terminally attached to the peptide sequence, **11** can also be radiolabeled with [^111^In]In^3+^, for imaging, or with [^90^Y]Y^3+^ and [^177^Lu]Lu^3+^, for the therapy of MTC. In this case, molecular modeling was used to determine whether CP04’s binding affinity for CCK-2R was sensitive to the type of radiometal complex and to obtain insights into the structure of the CP04–CCK2R complex.

**Figure 15:**
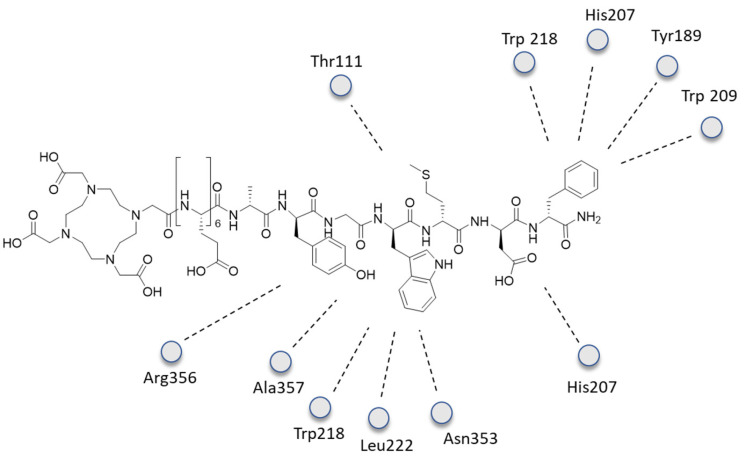
The 2D interactions of **11** inside the CCK2R receptor pocket.

Since no CCK-2R crystal structure has been reported so far, several conformations of the unlabeled **11** were generated and then docked to a CCK-2R retrieved from the homology model by using AutoDock Vina [34]. The binding box was selected to contain both the intrahelical cavity and the extracellular loops and then extended. The best-scored binding poses were manually inspected for compatibility with mutagenesis data reported for CCK derivatives binding to CCK-2R. Data from molecular docking showed that CP04 enters the CCK2R binding pocket with the *C*-terminus directed towards the intracellular side, creating numerous contacts between the peptide and the receptor (Figure 15). The aromatic side chain of the *C*-terminal Phe^1^ is in the “aromatic box” formed by Trp218^5.39^, His207^ECL2^, Tyr189^4.61^, and Trp209^ECL2^. The phenyl ring lies parallel to those of Trp218^5.39^ and His207^ECL2^ and perpendicular to Tyr189^4.61^. The charged side chain of the Asp^2^ points to His207^ECL2^. The positioning of the Met^3^ side chain is less tight. The side chain is oriented towards Thr111^2.60^, but as there is much free volume around it, multiple orientations probably co-exist. In the case of Trp^4^, the aromatic indole forms hydrophobic interactions with Trp218^5.39^, Leu222^5.43^, and Asn353^6.55^. A *π*–cation interaction is established between Arg356^6.58^ and the aromatic ring of Tyr^6^. Additional stabilization of the bound conformation may here arise from a hydrogen bond between the Tyr^6^ phenol function and the carbonyl of Ala357^6.59^.

According to this study, there is no significant difference in the binding of the compounds regarding whether they chelate different metals, even though ^Ga3+^-DOTA and ^Lu3+^-DOTA have different coordination structures.

Small changes in binding affinity regarding the different substitutions of the spacer, chelating moiety, and the presence/absence of metal, as well as its exact type, are understandable thanks to this binding model. According to the binding model, the *N*-terminal part of CP04 should be located at the receptor binding site exit, where there is a large free volume. This site is exposed to solvents; thus, the substructure may take different energetically similar conformations.

**Figure 16:**
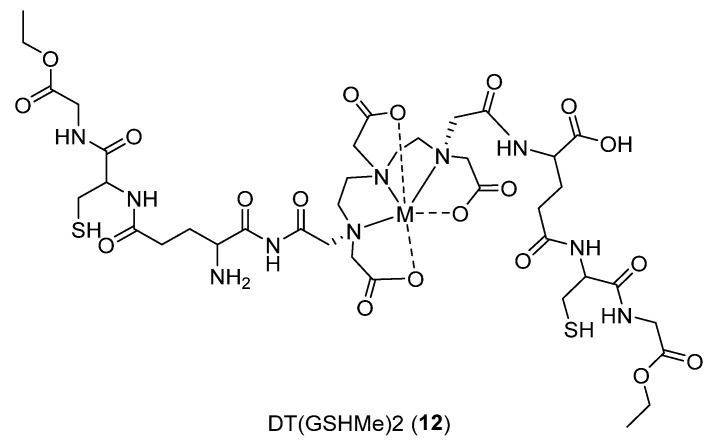
Structure of **12**.

Khurana, Harleen, et al. [35] aimed to design a novel probe for molecular imaging in proliferating with upregulated Gamma Glutamyl Transferase (GGT), named DT(GSHMe)2 **12** (Figure 16). GGT catalyzes the hydrolysis of extracellular *γ*-glutamyl compounds such as Glutathione (GSH) and leukotrienes and produces reactive oxygen species in malignant cells, causing tumor proliferation. The probe was prepared by conjugating DTPA with two GSH ethanol esters. Finally, coordinating with ^99m^Tc and ^68^Ga grants stable and safe radiochemistry with a cost-effective process.

Xenografted Ehrlich Ascites (EAT) cells and the cerebral glioma cell line (BMG-1) from a mice model show moderated and high GGT expression, respectively, evident from Western blotting. Kinetic studies show good transport into tumor cells, with fast clearance through the kidneys after initial accumulation in the blood, heart, and liver. Finally, the distribution rate is in favor of tumor cells. Thanks to the crystal structure of hGGT (PDB ID: 4GG2), docking studies have been performed using the Schrödinger software Maestro (Schrödinger, LLC, New York, NY, USA, 2014) for DT(GSHMe)2 and Re-DT(GSHMe)2. The first revealed one conformation with good binding affinity for the substrate, forming several hydrogen bond interactions. Conjugated metal complexes (Re and Tc) showed a 3D pose with Re-DT(GSHMe)2 in the substrate binding pocket at the unlabeled one. PET/SPECT imaging was performed on mice bearing xenografted tumors, revealing higher concentration of the probes in tumor cells.

Matrix metalloproteases (MMPs), a zinc-dependent proteolytic enzyme class, are associated with metastasis. In particular, MMP-14 has a critical regulatory role and is overexpressed in neuroblastoma, carcinoma, ovarian cancer, and breast cancer.

**Figure 17:**
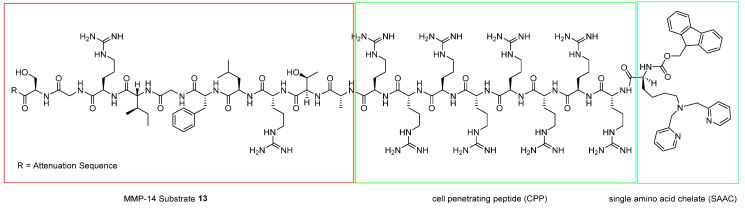
Structure of **13**.

**Figure 18:**
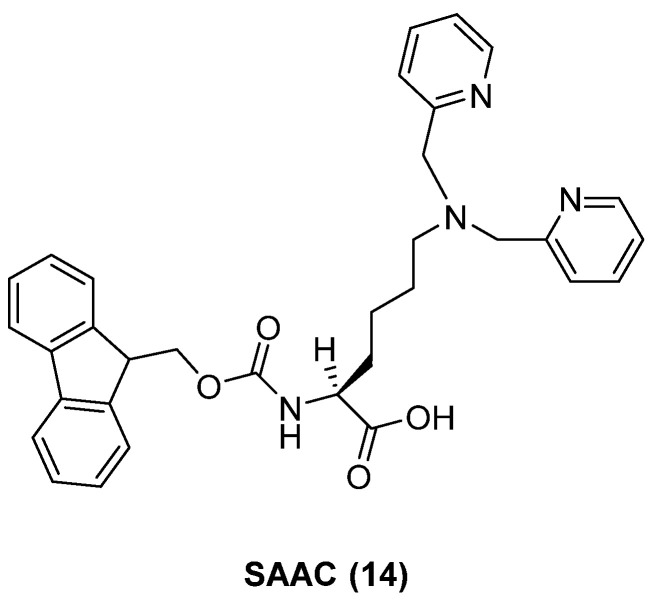
Structure of **14**.

The optimization of the activatable SPECT imaging probe targeting MMP-14 has been assessed through molecular modeling [36]. The designed MMP-14 probe (**13**) (Figure 17) is formed by a positively charged octamer (CCP) attacked with a single amino acid-chelated (SAAC) **14**, shown in Figure 18, for technetium-99m, an MMP-14-specific cleavable substrate and a negatively charged attenuating sequence.

A known sequence has been chosen as a cleavable substrate, whereas four attenuating sequences were synthesized. Molecular modeling was used to provide 3D conformation of probes and associate in silico results with the cleavage rate [37]. In vivo studies confirmed that the four d-glutamate-glycine-glycine repeat sequence has specific proteolytic activity in breast cancer (MDA-MB-231) cells transfected with MMP-14 cDNA to overexpress MMP-14. Computational research has proven to be a valuable tool in optimizing and, thus, realizing specific radiolabeled probes.

**Figure 19:**
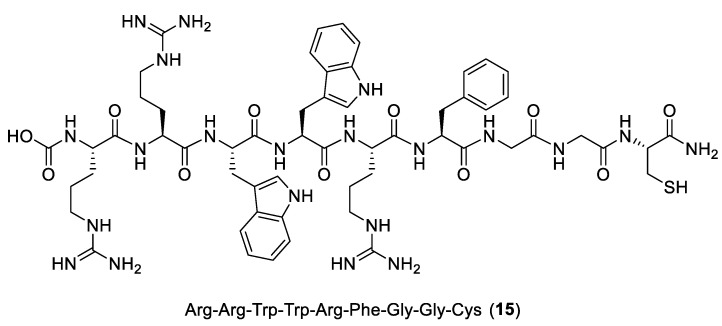
Structure of **15**.

In contrast to bacterial resistance, Kaul, Ankur, et al. [38] have rationally designed a radiolabeled nonapeptide **15** as an infection imaging agent from a bovine lactoferricin protein scanning hexapeptide combinatorial library (Figure 19). Peptide activity is given by interaction with the bacterial membrane and intracellular activity, distinguishing it from mammalian cells. In silico studies have been performed to estimate the net positive charge, hydrophobicity, and flexibility. The arginine and tryptophan of the sequence seem to play a vital role in the insertion into the cellular membrane. Arg forms salt bridges with phosphate groups, and Trp provides *π*–cation interactions. The crystal structure of *E. coli* PBP1b (PDB ID: 3VMA), a bifunctional peptidoglycan glycosyltransferase representing a transglycosylase’s structural platform, was selected for docking studies. The calculated binding affinity is mainly driven thanks to hydrogen bonds and electrostatic interaction, which have a key role in considering the negative charge of the bacterial membrane. The peptide was synthesized with solid resin and Fmoc chemistry. The following in vitro studies highlighted the activity against *E. coli*. Because of the fast clearance, the infectious lesion induced in a murine model was detected by in vivo gamma scintigraphy.

## 3. Conclusions

Peptide-based PET probes are very small tracers specifically designed to function as PET probes for diagnosis and/or therapy. They have high affinity and specificity for specific cellular targets. It is not unsurprising that the development of radiolabeled PET probes based on short peptides has attracted research for decades, given the critical role that peptides/cellular receptor interactions play. Compared to traditional probes based on antibodies, peptide-based probes exhibit several advantages. Peptides are less expensive and more suited for chemical site-specific modification to enable radiolabeling with any radionuclide [39,40]. These features make them particularly appealing for clinical applications. The radioisotope can be covalently coupled to the peptide probe, or the probe can function with another complexing agent to coordinate the radioisotope. This family of tracers is particularly appealing for the development of radiopharmaceuticals for the diagnosis and treatment of oncological diseases, due to the variety of labeling possibilities and techniques to tune and improve the pharmacokinetic properties of such molecules [41,42]. Computational modeling is a rapidly evolving field that has the potential to revolutionize molecular imaging in nuclear medicine. The rational design, structure-based design, and combinatorial library screening allow the optimization of the peptide sequence and modifications to improve the probes’ binding affinity, specificity, and pharmacokinetic properties. This review summarized the more recent and successful application of computational modeling to design radiolabeled peptides for PET and SPECT imaging applications. In future years, computational modeling will continue to affirm its position in pharmaceutical research [43]. Although CADD is still not widely used in PET probe design, as demonstrated by the low number of examples present in the literature, we are confident that it will have a relevant impact in the future. The pioneering work reported in this review could be an example of using these tools and facilitating the discovery process of such pharmaceuticals. Unfortunately, we must point out the limits of such approaches to truly answer the question, “has CADD impacted radiopharmaceutical drug design?”. It has potential but has not yet produced any noticeable effects. What have we learned from this? We believe that CADD is a promising strategy, especially when three-dimensional structural data are learned and exploited, but although the discussed studies in this review showed an improvement in the binding properties of such radiopharmaceuticals for their targets, unfortunately, other aspects of the pharmacokinetics may affect the real activity of the molecule—for example, alterations in serum binding, biodistribution, non-specific uptake, fast metabolism, and other chemical bio-modifications. The real failure is well proven by several examples discussed in the paper that showed poorly in vivo outcomes, and none of the discussed works reached clinical application. This unfortunate result aligns with data reporting that more than 90% of drug development fails, despite implementing many successful strategies [44]. Despite the well-proven ability to achieve better binding to the target, CADD still needs to be further developed to simulate the real biological environment better and, in this way, minimize the high failure rate of today’s tools.

## Data Availability

Not applicable.

## Abbreviations

ABPP	Aβ binding peptide probe
AD	Alzheimer’s disease
Ahx	6-aminohexanoic acid
Aβ42	amyloid-beta 42
BB2R	bombesin receptor 2
BB3	bombesin receptor 3
BCa	breast cancer
BMG-1	cerebral glioma cell line
CADD	computer-aided drug design
CCK	cholecystokinin receptors
CCK-2R	cholecystokinin receptor subtype 2
CCP	positively charged octamer
cDNA	complementary DNA
CP04	minigastrin analog DOTA-(dGlu)-6-Ala-Tyr-Gly-Trp-Met-Asp-Phe-NH_2_
CSF	cerebral-cerebrospinal fluid
C-X-C	chemokine receptors
CXCR4	chemokine receptor 4
DBCO	dibenzocyclooctyne
DCIBzL	((1-carboxy-5-(4-iodobenzamido)pentyl)carbamoyl)glutamic acid
DOTA	1,4,7,10-tetraazacyclododecane-1,4,7,10-tetraacetic acid
DOTATOC	1,4,7,10-tetraazacyclododecan-4,7,10-tricarboxy-meth-1-yl-acetyl-d-Phe-Tyr3-octreotide
DTPA	pentetic acid, diethylenetriaminepentaacetic acid
EAT	Xenografted Ehrlich Ascites
ELISA	enzyme-linked immunosorbent assay
ESCC	esophageal squamous cell carcinoma
Fmoc	fluorenylmethoxycarbonyl protecting group
GCP II	glutamate carboxypeptidase II
GGT	gamma glutamyl transferase
GRPR	gastrin releasing peptide receptor
GSH	glutathione
HBED-CC	*N*,*N*′-bis-[2-hydroxy-5-(carboxyethyl)benzyl]ethylenediamine-*N*,*N*-diacetic acid
HIV-1	human immunodeficiency virus
MD	molecular dynamics
MMPs	matrix metalloproteases
MOLCAD	Computer-Aided Visualization and Manipulation of Models
MTC	medullary thyroid carcinoma cells
NOTA	2,2′,2″-(1,4,7-triazacyclononane-1,4,7-triyl)triacetic acid
NOTA-CP01	cyclo[Phe-Tyr-Lys-(*i*Pr)-d-Arg-2-Nal-Gly-d-Glu]-Lys-(*i*Pr)-butane-1,4-diamine-bis-*t*-butyl NOTA-NH_2_
PCa	prostate cancer
PEG	polyethylene glycol
PET	positron emission tomography
PSMA	prostate-specific membrane antigen
RM2	gastrin-releasing peptide receptor antagonist
S1	non-prime section
S1′	prime section
SAAC	single amino acid chelated
SAR	structure–activity relationship
SDF-1/CXCL12	stromal cell-derived factor 1
Siglec-9	sialic acid-binding Ig-like lectin 9
SPECT	single-photon emission computed tomography
SSTR2	somatostatin receptor subtype 2
TE1K1P−PEG4−DBCO−Y3-TATE	
TRNT	targeted radionuclide therapy
VAP-1	vascular adhesion protein-1
Y3-TATE	tyrosine 3-octreotate
